# Combined maternal central adiposity measures in relation to infant birth size

**DOI:** 10.1038/s41598-024-51274-6

**Published:** 2024-01-06

**Authors:** Emelie Lindberger, Fredrik Ahlsson, Katja Junus, Anna-Karin Wikström, Inger Sundström Poromaa

**Affiliations:** Department of Women’s and Children’s Health, Uppsala University, 751 85 Uppsala, Sweden

**Keywords:** Intrauterine growth, Ultrasonography, Pregnancy outcome

## Abstract

Improvement of prenatal identification of large-for-gestational-age (LGA) infants could lower the risk for adverse outcomes. Therefore, we sought to evaluate the association of a combination of maternal waist circumference (WC) and abdominal fat depths with infant birth size. A cohort study including 1240 women was performed between 2015 and 2018 at Uppsala University Hospital, Sweden. Maternal WC was measured at the first antenatal visit, and visceral (VF) and subcutaneous (SCF) fat depths by ultrasound at the second-trimester anomaly scan. Waist circumference, VF, and SCF were categorized as low or high (cut-offs WC ≥ 88 cm, VF ≥ 54 mm, SCF ≥ 21 mm). Outcomes were birth weight standard deviation score (BWSDS) and LGA (BWSDS > 90th and > 97th percentile). Secondary outcome was small-for-gestational-age (SGA, BWSDS < 10th and < 3rd percentile). Univariate analysis of variance and logistic regression analyses were performed adjusted for maternal weight, height, parity, smoking, country of birth, pregestational diabetes, and chronic hypertension. For both high and low WC, high VF was positively associated with BWSDS and LGA. There was no association with SGA. The results did not demonstrate any value of the combination of WC and fat depth measures in predicting infant birth size but suggested VF as a marker for large infants.

## Introduction

Being born large-for-gestational-age (LGA), often defined as a birthweight > 90th percentile after adjustments for gestational age and sex^1^, is associated with adverse pregnancy outcomes for both the mother and the infant. For the mother, giving birth to an LGA-infant increases the risk for emergency caesarian section, perineal injuries, and postpartum bleeding^2^. The LGA-infant is at increased risk of shoulder dystocia, plexus brachialis injury^2^, low Apgar score^3^, and neonatal hypoglycemia^4^. Being born LGA might also have long-term consequences, as it is associated with malignancies in childhood^5^, obesity^6^, diabetes mellitus^5,6^, breast cancer, cardiovascular disease, and psychiatric disorders^5^.

Several maternal factors are associated with increased fetal growth and high birth weight, such as overweight and obesity, excessive pregnancy weight gain^7^, and diabetes mellitus^8,9^. Previous studies have suggested that prenatal identification of LGA-fetuses, which enables appropriate interventions at delivery, could lower the risk for adverse outcomes^3,10^. Hence, early detection of LGA-infants is of high clinical importance, and improving methods for early identification of LGA-fetuses is necessary.

Maternal BMI is a recognized predictor of infant birth weight and is used in routine care for risk stratification of pregnant women. However, BMI may be insufficient as an individual risk predictor because it does not directly estimate adiposity^11^. In fact, BMI has high specificity but low sensitivity in identifying individuals with excess body fat; only 50% of non-pregnant individuals with adiposity-related risk are identified by BMI^12^. Waist circumference (WC) is suggested as an independent risk marker of cardiometabolic complications due to its ability to target individuals with central adiposity, i.e. increased visceral fat mass. Waist circumference measurement is recommended to identify non-pregnant individuals with the highest risk of obesity-related complications^13^.

Maternal WC correlates with infant birth size^14,15^, and an independent association between early mid-pregnancy visceral fat depth and birth weight has previously been reported by our group^16^. We hypothesize that a combination of early pregnancy WC and early mid-pregnancy fat depth measures, especially visceral fat, could be used to predict increased infant birth weight. This population-based cohort study, including 1240 women and child-dyads, sought to evaluate the value of the combination of early pregnancy WC and early mid-pregnancy ultrasound estimated abdominal fat depths in predicting infant birth size.

## Methods

### Study population

From January 2015 to December 2017, WC measurement was implemented as a clinical routine at the first antenatal visit in Uppsala County, Sweden. A total of 5827 pregnant women underwent WC measurement during this period. In addition, measurement of visceral fat depth (VF) and subcutaneous fat depth (SCF) was implemented as a clinical routine at the second-trimester anomaly scan at Uppsala University Hospital, Sweden. It was a coincidence whether the scan included fat depth measurement since the personnel booking the scans was not involved in the study. During the study period, 2844 women underwent a scan including fat depth measurements^17^. Eligible participants for this cohort study were women who had undergone both WC and fat depth measurements (n = 1366).

A research database including WC and fat depth measurements was created. Information on maternal age, maternal weight at the first antenatal visit, maternal height, smoking at the first antenatal visit, maternal country of birth, chronic illness, infant birth weight, gestational age, and sex was linked from the Medical Birth Register held by the Swedish National Board of Health and Welfare. Following linkage, the study population database was pseudo-anonymized.

### Exposures

Waist circumference was measured with a standardized measurement tape between the lower rib margin and the iliac crest with the woman standing^18^. The midwives who measured WC received verbal and written information repeatedly during the study period on how to perform measurements. We defined a low WC measure as < 88 cm and a high as ≥ 88 cm. This cut-off was selected based on the WHO-suggested WC cut-off for (non-pregnant) women, where WC ≥ 88 cm implies a substantially increased risk for metabolic complications associated with obesity^18^. In addition, this WC cut-off has been used by others in the same research field^19,20^.

Maternal VF and SCF were measured at the second-trimester anomaly scan at 18–19 weeks’ gestation, as first described by Armellini et al.^21^, with a minor adjustment regarding the placement of the probe. The measurements were performed with a GE Voluson E6, E8, or E10 ultrasound machine (GE Medical Systems, Zipf, Austria) with the woman in the supine position. The ultrasound probe was placed at the body’s midline 10 cm above the umbilicus. The VF was defined as the distance between the inner border of the rectus abdominis muscle and the anterior border of the aorta, and the SCF was defined as the distance between the dermis and the surface of the rectus abdominis muscle. Both fat depths were measured in millimeters. All midwives who performed the measurements were certified obstetric ultra-sonographers. Additional training sessions were held throughout the study period to maximize the scan quality. The intraclass correlation coefficient of the inter-examiner variation was 0.83 for VF and 0.85 for SCF, indicating good reliability^22^. Visceral fat depth and SCF were categorized in quartiles (VF quartile 1–4 and SCF quartile 1–4). Quartile 4 was defined as high (VF ≥ 54 mm and SCF ≥ 21 mm), and quartiles 1–3 as low (VF < 54 mm and SCF < 21 mm).

### Main outcomes

Infant birth size was evaluated as birth weight standard deviation score (BWSDS) and LGA. The BWSDS is a population-based z-score and was calculated by the use of national reference standards for birth weight with respect to gestational age and sex^23^. We used two definitions of LGA: BWSDS above the 90th percentile and BWSDS above the 97th percentile (used as a proxy for + 2 standard deviations, the clinical definition of LGA used in Sweden). We also evaluated small-for-gestational-age (SGA) as a secondary outcome. Two definitions of SGA were used: BWSDS below the 10th percentile and BWSDS below the 3rd percentile (used as a proxy for − 2 standard deviations, the clinical definition of SGA used in Sweden).

### Ethical approval

The study was approved by the Regional Ethical Review Board in Uppsala (Dnr: 2014/353 and 2015/366). All research was performed in accordance with relevant national and international guidelines. Informed consent was waived by the Swedish Ethical Review Authority (Dnr: 2019–00391).

### Statistics

The Welch ANOVA test, followed by the Games-Howell post-hoc test, was used to evaluate WC, VF, and SCF in relation to BMI classes since the assumption of homogeneity of variances was not met. Pearson correlation coefficients were calculated to examine the associations between BMI, WC, VF, and SCF.

To evaluate the value of different combinations of central adiposity measures in predicting infant birth size, the cohort was divided into eight groups based on WC and fat depths (Table 1). The low-risk group (low WC/low VF/low SCF) was the reference. Univariate analysis of variance was performed to evaluate the difference in BWSDS between the low-risk group and the other seven groups. The analysis was adjusted for maternal weight at the first antenatal visit (kg), maternal height (cm), parity (nulliparous or parous), smoking at the first antenatal visit (yes or no), maternal country of birth (EU or outside EU), pregestational diabetes (yes or no), and chronic hypertension (yes or no). Covariates were selected based on previous prediction models for large infants^24,25^.

**Table 1:** Groups based on waist circumference and fat depth measures.

Combination of measurements	n (%)
Low WC/low VF/low SCF (ref.)	658 (53.1)
Low WC/high VF/low SCF	120 (9.7)
Low WC/low VF/high SCF	99 (8.0)
Low WC/high VF/high SCF	30 (2.4)
High WC/low VF/low SCF	76 (6.1)
High WC/high VF/low SCF	54 (4.4)
High WC/low VF/high SCF	96 (7.7)
High WC/high VF/high SCF	107 (8.6)

Low WC < 88 cm; high WC ≥ 88 cm; low VF < 54 mm; high VF ≥ 54 mm; low SCF < 21 mm; high SCF ≥ 21 mm.

WC, waist circumference; VF, visceral fat depth; SCF, subcutaneous fat depth.

Logistic regression was used to evaluate differences in the odds of giving birth to an LGA or SGA infant between the low-risk group and the other seven groups. The same covariates as described above were included in the models. All data were analyzed using IBM SPSS Statistics version 28. The statistical significance level was set at *p* < 0.05.

## Results

Out of 1366 women with both WC and fat depth measurements, 126 women were excluded from further analysis due to missing information on BMI at first antenatal visit (n = 11), multiple pregnancy (n = 2), intrauterine fetal death (n = 3), missing data from the delivery (n = 30), WC measurement not from current pregnancy (n = 32), WC measured < 5 or > 16 weeks’ gestation (n = 45), and error value of BMI, VF or SCF measure (n = 3). The final study population consisted of 1240 women who gave birth to singleton infants between 8 July 2015 and 2 September 2018. The women were 15–45 years old, 559 (45.1%) were nulliparous, and 515 (41.5%) were either overweight or obese. A WC ≥ 88 cm was observed in 333 women (26.9%). The study population characteristics are presented in Table 2.

**Table 2:** Descriptive characteristics of the study population.

	Variable	Cohort
Women	n	1240
	Age, years (mean ± SD)	30.2 ± 4.8
	Weight, kg (mean ± SD)	69.7 ± 14.4
	Height, cm (mean ± SD)	166.5 ± 6.3
	Nulliparous, n (%)	559 (45.1)
	Smoking at first antenatal visit^a^, n (%)	43 (3.5)
	Country of birth within the EU, n (%)	1066 (86.0)
	Pregestational diabetes mellitus, n (%)	5 (0.4)
	Essential hypertension, n (%)	2 (0.2)
	Early pregnancy BMI kg/m^2^ (mean ± SD)	25.1 ± 4.8
	BMI < 18.5 kg/m^2^ (underweight), n (%)	28 (2.3)
	BMI 18.5 to 24.9 kg/m^2^ (normal weight), n (%)	697 (56.2)
	BMI 25.0 to 29.9 kg/m^2^ (overweight), n (%)	325 (26.2)
	BMI ≥ 30.0 kg/m^2^ (obesity), n (%)	190 (15.3)
	Waist circumference ≥ 88 cm, n (%)	333 (26.9)
	Gestational diabetes mellitus, n (%)	29 (2.3)
Infants	n	1240
	Gestational length, days (mean ± SD)	279 ± 12
	Preterm, n (%)	53 (4.3)
	Post-term, n (%)	76 (6.1)
	Birth weight, g (mean ± SD)	3554 ± 529
	SGA < 10th percentile, n (%)	108 (8.7)
	SGA < 3rd percentile, n (%)	32 (2.6)
	LGA > 90th percentile, n (%)	142 (11.5)
	LGA > 97th percentile, n (%)	68 (5.5)

SD, standard deviation; BMI, body mass index; preterm, < 37 ^+0^ weeks’ gestation; post-term, > 41 ^+6^ weeks’ gestation; SGA, small-for-gestational-age (birth weight standard deviation score (BWSDS) below the 10^th^ percentile or 3rd percentile); LGA, large-for-gestational-age (BWSDS above the 90^th^ percentile or 97^th^ percentile).

^a^ Information on smoking status was missing in 4.4% of study participants.

### Waist circumference and fat depth measures in relation to BMI

The WC measurements were obtained at a mean of 64 days’ gestation (standard deviation (SD) 15 days). The vast majority (91%) of the women had their WC measured < 12 weeks’ gestation. Overall, WC ranged 60–150 cm, VF 2–108 mm, and SCF 1–52 mm. Waist circumference, VF, and SCF measures in relation to WHO BMI classes are presented in Supplementary Table S1. All three adiposity measures increased with increasing BMI (Supplementary Fig. S1, panel A–C).

Waist circumference and BMI were highly correlated (r = 0.84)^26^ (Supplementary Table S2). A low correlation was seen between VF and BMI (r = 0.44), whereas SCF and BMI were moderately correlated (r = 0.67) (Supplementary Table S2). There was a low correlation between WC and VF (r = 0.41) and a moderate correlation between WC and SCF (r = 0.62) (Supplementary Table S2).

### Associations of the combination of WC and fat depths with BWSDS

In comparison with the low-risk group, increased BWSDS was observed in the low WC/high VF/low SCF group (mean difference 0.23, CI 0.03 to 0.44, *p* = 0.025), and in the high WC/high VF/low SCF group (mean difference 0.42, CI 0.10 to 0.73, *p* = 0.010) (Table 3).

**Table 3:** Association of the combination of WC and fat depth measures with infant birth weight standard deviation score (BWSDS), pairwise comparison.

Combination of measurements	Mean difference in BWSDS	CI	*p*
Low WC/low VF/low SCF (ref.)			
**Low WC/high VF/low SCF**	**0.23**	**0.03 to 0.44**	**0.025**
Low WC/low VF/high SCF	0.08	− 0.15 to 0.31	0.499
Low WC/high VF/high SCF	0.38	0.00 to 0.77	0.052
High WC/low VF/low SCF	0.05	− 0.22 to 0.31	0.734
**High WC/high VF/low SCF**	**0.42**	**0.10 to 0.73**	**0.010**
High WC/low VF/high SCF	− 0.13	− 0.41 to 0.15	0.351
High WC/high VF/high SCF	0.29	− 0.01 to 0.59	0.057

BWSDS, birth weight standard deviation score; low WC < 88 cm; high WC ≥ 88 cm; low VF < 54 mm; high VF ≥ 54 mm; low SCF < 21 mm; high SCF ≥ 21 mm.

WC, waist circumference; VF, visceral fat depth; SCF, subcutaneous fat depth; OR, odds ratio; CI 95% confidence interval.

Data were analyzed using univariate analysis of variance. Bold text indicates a statistically significant result.

The analyses were adjusted for maternal weight at the first antenatal visit (kg), maternal height (cm), parity (nulliparous or parous), smoking at the first antenatal visit (yes or no), maternal country of birth (EU or outside EU), pregestational diabetes (yes or no), and chronic hypertension (yes or no).

### Associations of the combination of WC and fat depths with LGA and SGA

In comparison with the low-risk group, there was an increase in the odds of giving birth to an infant LGA (defined as BWSDS > 90th percentile) in the following groups: low WC/high VF/low SCF (odds ratio (OR) 2.20, CI 1.21 to 4.05, *p* = 0.010), low WC/high VF/high SCF (OR 4.04, CI 1.58 to 10.31, *p* = 0.004), high WC/high VF/low SCF (OR 2.47, CI 1.11 to 5.50, *p* = 0.027), and high WC/high VF/high SCF (OR 2.40, CI 1.06 to 5.44, *p* = 0.037) (Table 4). In the analyses evaluating the stricter definition of LGA (BWSDS > 97th percentile), the odds were increased in the same groups, and the estimates were higher (Table 5). In the analyses evaluating SGA (BWSDS < 10th percentile and < 3rd percentile), there were no significant results (Supplementary Table S3 and Supplementary Table S4).

**Table 4:** Association of the combination of WC and fat depth measures with LGA defined as birth weight standard deviation score > 90th percentile.

Combination of measurements	LGA, n (%)	OR	CI	*p*
Low WC/low VF/low SCF (ref.)	49 (7.4)	1.00		
**Low WC/high VF/low SCF**	**18 (15.0)**	**2.20**	**1.21 to 4.05**	**0.010**
Low WC/low VF/high SCF	9 (9.1)	1.21	0.54 to 2.70	0.639
**Low WC/high VF/high SCF**	**7 (23.3)**	**4.04**	**1.58 to 10.31**	**0.004**
High WC/low VF/low SCF	9 (11.8)	1.07	0.46 to 2.48	0.877
**High WC/high VF/low SCF**	**12 (22.2)**	**2.47**	**1.11 to 5.50**	**0.027**
High WC/low VF/high SCF	14 (14.6)	1.37	0.60 to 3.11	0.457
**High WC/high VF/high SCF**	**24 (22.4)**	**2.40**	**1.06 to 5.44**	**0.037**

LGA, large-for-gestational-age; low WC < 88 cm; high WC ≥ 88 cm; low VF < 54 mm; high VF ≥ 54 mm; low SCF < 21 mm; high SCF ≥ 21 mm.

WC, waist circumference; VF, visceral fat depth; SCF, subcutaneous fat depth; OR, odds ratio; CI 95% confidence interval.

Data were analyzed using logistic regression. Bold text indicates a statistically significant result.

The analyses were adjusted for maternal weight at the first antenatal visit (kg), maternal height (cm), parity (nulliparous or parous), smoking at the first antenatal visit (yes or no), maternal country of birth (EU or outside EU), pregestational diabetes (yes or no), and chronic hypertension (yes or no).

**Table 5:** Association of the combination of WC and fat depth measures with LGA defined as birth weight standard deviation score > 97th percentile.

Combination of measurements	LGA, n (%)	OR	CI	*p*
Low WC/low VF/low SCF (ref.)	15 (2.3)	1.00		
**Low WC/high VF/low SCF**	**10 (8.3)**	**3.91**	**1.66 to 9.18**	**0.002**
Low WC/low VF/high SCF	5 (5.1)	2.39	0.82 to 6.94	0.109
**Low WC/high VF/high SCF**	**4 (13.3)**	**6.53**	**1.92 to 22.15**	**0.003**
High WC/low VF/low SCF	3 (3.9)	1.08	0.27 to 4.27	0.914
**High WC/high VF/low SCF**	**8 (14.8)**	**4.68**	**1.65 to 13.23**	**0.004**
High WC/low VF/high SCF	9 (9.4)	2.34	0.77 to 7.09	0.132
**High WC/high VF/high SCF**	**14 (13.1)**	**3.46**	**1.13 to 10.56**	**0.030**

LGA, large-for-gestational-age; low WC < 88 cm; high WC ≥ 88 cm; low VF < 54 mm; high VF ≥ 54 mm; low SCF < 21 mm; high SCF ≥ 21 mm.

WC, waist circumference; VF, visceral fat depth; SCF, subcutaneous fat depth; OR, odds ratio; CI 95% confidence interval.

Data were analyzed using logistic regression. Bold text indicates a statistically significant result.

The analyses were adjusted for maternal weight at the first antenatal visit (kg), maternal height (cm), parity (nulliparous or parous), smoking at the first antenatal visit (yes or no), maternal country of birth (EU or outside EU), pregestational diabetes (yes or no), and chronic hypertension (yes or no).

## Discussion

This is, to the best of our knowledge, the first study to evaluate the value of a combination of early pregnancy WC and early mid-pregnancy ultrasound estimated fat depths in predicting infant birth size. Infants of mothers with a high VF, regardless of WC and SCF measures, had higher odds of LGA compared with infants of mothers in the low-risk group. Additionally, the BWSDS was higher among infants of mothers with high VF and low SCF, independent of WC. These findings indicate that VF is a stronger predictor for large infants than WC or SCF. Interestingly, the highest odds of LGA were observed among women with low WC and high fat depth measures. This group might include women with an unhealthy metabolic profile despite a low WC. Women with a low WC are likely to have a low BMI since these measures are highly correlated. From a clinical perspective, using ultrasound to detect VF might have the greatest value in women with low BMI, as these are not otherwise identified as at risk.

Our results are in line with a previous study reporting a positive association of first trimester VF, but not WC, with birth weight centile^27^. Other previous studies have studied either WC or VF as proxies for central adiposity in relation to infant outcomes. Of seven previous studies evaluating the association between WC and infant birth size, six report a positive association with birth weight, LGA, and macrosomia (≥ 4000 g)^14,15,20,28–30^. These results are different from ours, we did not find any association between WC and birth size when a combination of central adiposity measures was evaluated. One could speculate that WC might predict large infants in a model not including VF. When both measures are available, as in our study, VF seems to be superior to WC. Our results are also supported by the findings of three previous studies reporting a positive association between maternal VF, measured by ultrasound, and infant birth size^16,27,31^.

We used ultrasound to measure the fat depths, but the gold standard methods for examination of intra-abdominal fat mass are CT and MRI^32^. However, for abdominal fat distribution assessment during pregnancy, ultrasound is a more feasible method for several reasons. First, pregnant women are already being examined by a trained ultrasonographer at the routine antenatal ultrasound scan, and fat depth measurements could easily be implemented at this time point with no need for further health care visits. Second, ultrasound is more accessible and less expensive than CT and MRI. Third, ultrasound does not involve any radiation, which could be harmful to the fetus. Abdominal ultrasound is a reliable and reproducible method for examination of intra-abdominal fat mass; a correlation coefficient of 0.81 (*p* < 0.001) between ultrasound and CT measures has been reported, indicating a strong association^33^.

Waist circumference measurement also has advantages. It is a cheap, easy, and fast method for body fat distribution assessment that could be implemented in routine care of pregnant women, especially in low-resource settings. However, we only found a weak correlation between early pregnancy WC and early mid-pregnancy VF, indicating that WC is not a good proxy for visceral fat accumulation in pregnant women.

The possible causal pathways linking maternal visceral fat accumulation to increased birth weight are not fully elucidated, but insulin resistance and hyperglycemia could be partly responsible. Early pregnancy visceral fat thickness correlates positively with diastolic blood pressure and levels of insulin, blood glucose, triglycerides, and cholesterol^34^. The physiology of normal pregnancy includes peripheral insulin resistance and hyperlipidemia^35^, and excessiveness of these normal metabolic changes among pregnant women with central obesity could possibly underpin the association between visceral fat and high birth weights.

This cohort study had strengths and limitations. Limitations included that the number of study participants was small in some of the groups, which might have lowered the power of the study. Yet another limitation was that the WC and fat depths were measured only once. A strength was that the study cohort was population-based. To ensure that the WC measure belonged to the current pregnancy and to avoid the impact of the growing uterus, we only included women with a WC measure obtained > 5 and < 16 weeks’ gestation (91% had their WC measured < 12 weeks’ gestation). This range is applicable since there is no relation between WC measured at 6‒16 weeks’ gestation and gestational length^36^. In addition, WC is considered to be generally unaffected by the pregnancy until 20 weeks’ gestation, when the uterus reaches the umbilical level^37^. Furthermore, the timing of the VF measurement in our study (18‒19 weeks’ gestation) was unlikely prone to bias, as there is no significant difference in visceral fat thickness between the first trimester (8–12 weeks’ gestation) and the second trimester (24–27 weeks’ gestation)^38^. Yet another strength of this study was the outcome birth size. Birth weight was measured at the hospital in a standardized way soon after delivery. It was also beneficial that we used a standardized score taking gestational length and infant sex into account, which otherwise could have biased the results.

## Conclusions

This study did not show any predictive value of the combination of WC and fat depth measures on increased birth size but suggested VF as a marker for this outcome. Further studies are required to confirm our results. It could be valuable to evaluate possible cut-off points for VF, perhaps with respect to BMI classes, in relation to outcomes. The adiposity measure VF might improve the ability to identify pregnant women with the most hazardous obesity phenotype and to target health care interventions to those with the greatest risk for complications.

### Supplementary Information

Supplementary Information.

## Data Availability

The datasets generated during and/or analyzed during the current study are available from the corresponding author on reasonable request.
