# Supplementary material for: Combined maternal central adiposity measures in relation to infant birth size
Source: Sci Rep. 2024 Jan 6;14:725. doi: 10.1038/s41598-024-51274-6 (PMC10771412; doi:10.1038/s41598-024-51274-6)
Supplement: Supplementary file 1 — Supplementary Information. [file 41598_2024_51274_MOESM1_ESM.pdf]

Supplementary information file

Combined maternal central adiposity measures in relation to infant birth size

Emelie Lindberger MD PhD[1*], Fredrik Ahlsson MD PhD[1], Katja Junus MD PhD[1], Anna-Karin Wikström MD PhD[1], Inger Sundström Poromaa MD PhD[1]

[1] Department of Women's and Children's Health, Uppsala University, 751 85 Uppsala, Sweden

Supplementary Table S1. Waist circumference, visceral fat, and subcutaneous fat measures in relation to BMI classes defined by the World Health Organization.

**BMI, WHO classification (kg/m^2)**		**Waist circumference (cm)**		**Visceral fat (mm)**		**Subcutaneous fat (mm)**	
	**n**	**Mean ± SD**	**Min – max**	**Mean ± SD**	**Min – max**	**Mean ± SD**	**Min – max**
<18.5 (Underweight)	28	68 ± 6[a]	60 – 84	33 ± 11[e]	11 – 57	9 ± 4[a]	3 – 17
18.5 – 24.9 (Normal weight)	697	77 ± 6 [a]	60 – 97	40 ± 14[f]	2 – 83	14 ± 5[a]	1 – 39
25.0 – 29.9 (Overweight)	325	86 ± 7 [a]	63 – 110	47 ± 16[g]	5 – 85	19 ± 6[k]	2 – 46
30.0 – 34.9 (Obesity class I)	136	97 ± 7[b]	81 – 119	57 ± 16[h]	18 – 108	25 ± 7[l]	6 – 45
35.0 – 39.9 (Obesity class II)	41	106 ± 8[c]	90 – 124	59 ± 13[i]	37 – 94	30 ± 7[m]	13 – 44
≥40 (Obesity class III)	13	116 ± 15[d]	98 – 150	65 ± 18[j]	32 – 83	32 ± 8[n]	22 – 52

Welch ANOVA followed by Games-Howell post-hoc test.

[a] different from all other WHO BMI-classes, *P* <0.001.

[b] different from all other WHO BMI-classes, *P* =0.01 for obesity class III and *P* <0.001 for all other

[c] different from all other WHO BMI-classes (except obesity class III), *P* <0.001.

[d] different from all other WHO BMI-classes (except obesity class II), *P* =0.01 for obesity class I, *P* <0.001 for all other.

[e] different from all other WHO BMI-classes (except normal weight), *P* <0.001.

[f] different from all other WHO BMI-classes (except underweight), *P* <0.01 for obesity class III, *P* <0.001 for all other

[g] different from all other WHO BMI-classes, *P* <0.05 for obesity class III and *P* <0.001 for all other

[h] different from all other WHO BMI-classes (except obesity class II and III), *P* <0.001.

[i] different from all other WHO BMI-classes (except obesity class I and III), *P* <0.001.

[j] different from all other WHO BMI-classes (except obesity class I and II), *P* <0.05 for overweight, *P* <0.01 for normal weight, and *P* <0.001 for underweight.

[k] different from all other WHO BMI-classes, *P* =0.001 for obesity class III and *P* <0.001 for all other

[l] different from all other WHO BMI-classes (except obesity class III), *P* <0.01 for obesity class II, *P* <0.001 for all other.

[m] different from all other WHO BMI-classes (except obesity class III), *P* <0.01 for obesity class I, *P* <0.001 for all other.

[n] different from all other WHO BMI-classes (except obesity class I and II), *P* =0.001 for overweight and *P* <0.001 for all other.

BMI, body mass index; SD, standard deviation.

Supplementary Figure S1. Scatter plots with fit line showing: a) waist circumference (WC) vs body mass index (BMI); b) visceral fat depth (VF) vs BMI; and c) subcutaneous fat depth (SCF) vs BMI.

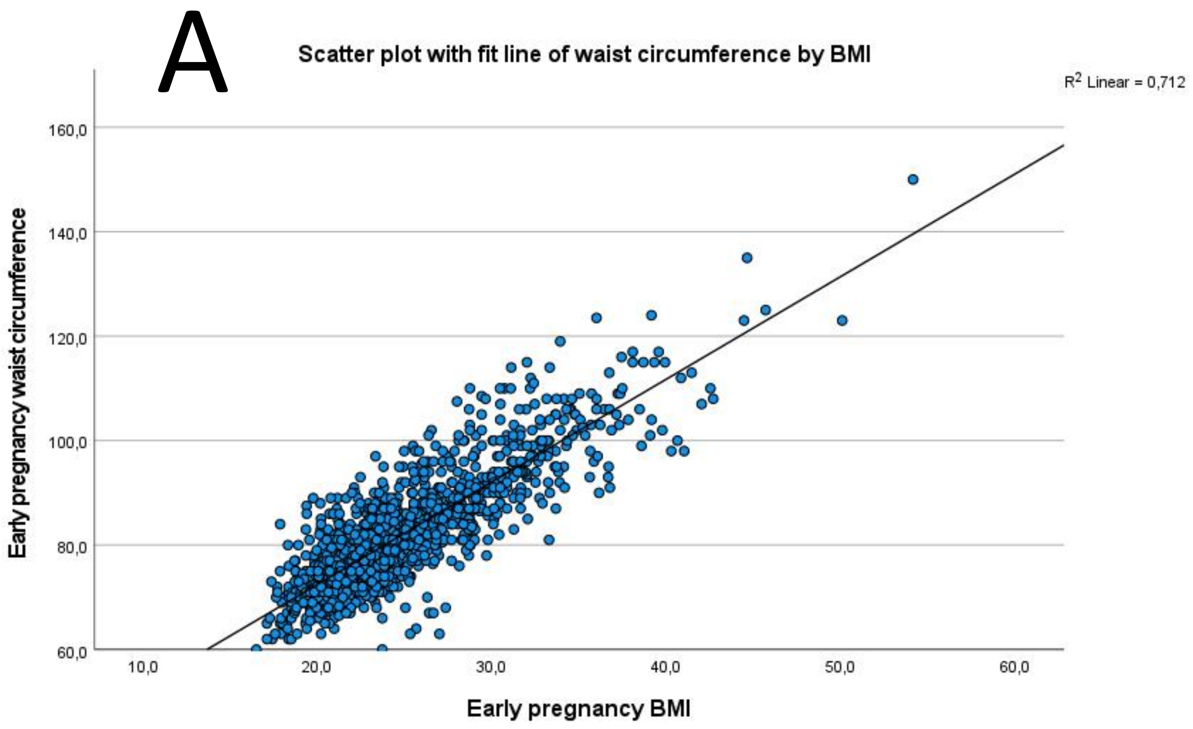

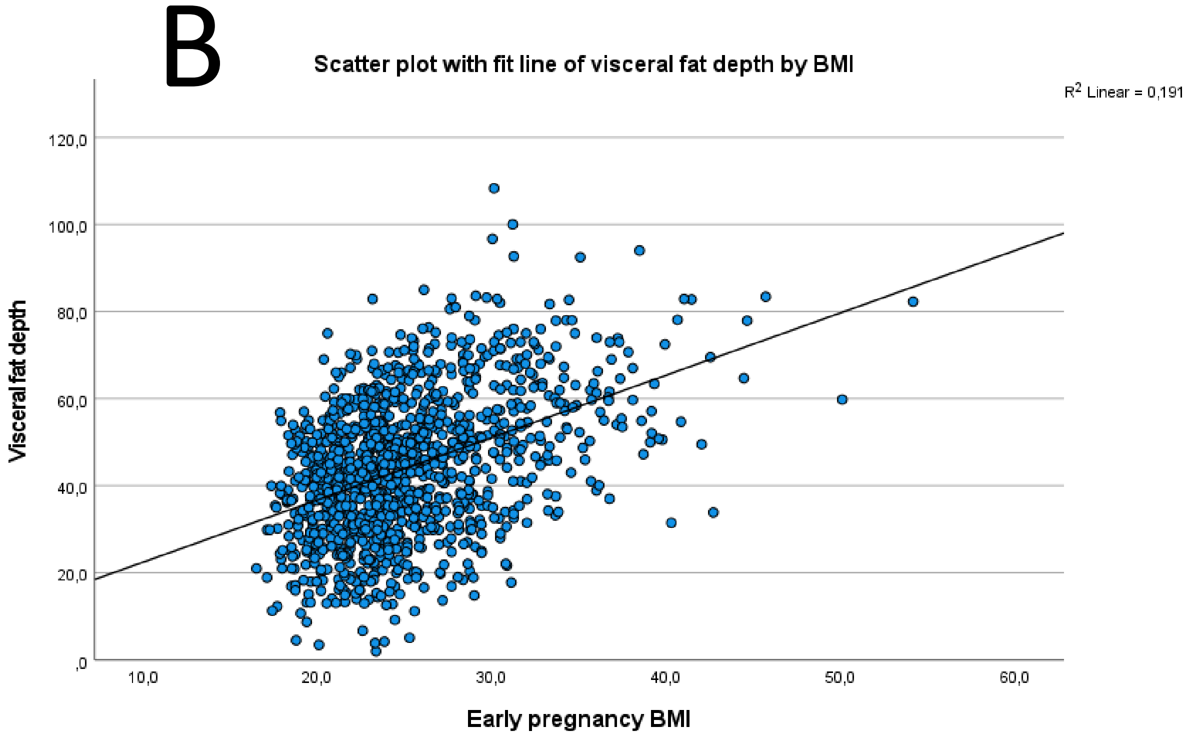

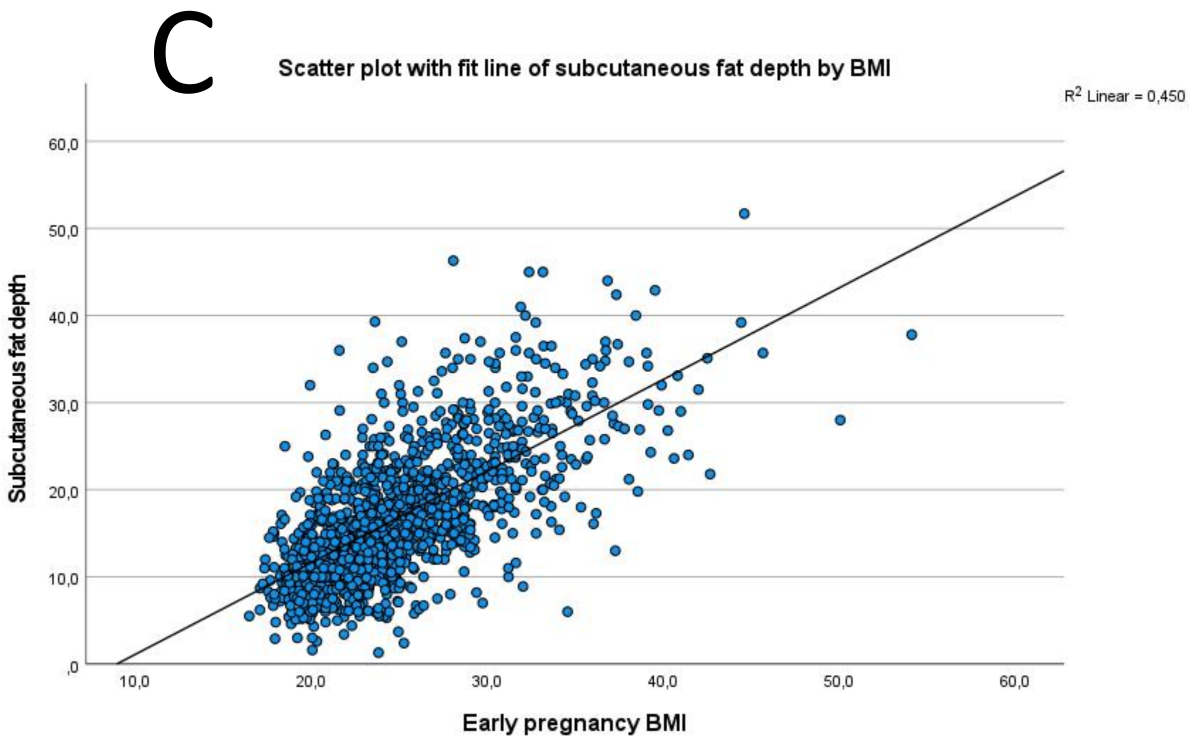
C
Scatter plot with fit line of subcutaneous fat depth by BMI
R2 Linear = 0,450
60,0
50,0
40,0
30,0
20,0
10,0
,0
Subcutaneous fat depth
10,0
20,0
30,0
40,0
50,0
60,0
Early pregnancy BMI

Supplementary Table S2. Correlation coefficients for the linear associations between waist circumference, fat depth measures, and BMI.

	**Early pregnancy BMI**	**Waist circumference**	**Visceral fat**	**Subcutaneous fat**
**Early pregnancy BMI**		0.84**	0.44**	0.67**
**Waist circumference**			0.41**	0.62**
**Visceral fat**				0.28**

BMI, body mass index.

Data are Pearson correlation coefficients.

** P <0.01.

Supplementary Table S3. Association of the combination of WC and fat depth measures with SGA defined as birth weight standard deviation score <10th percentile.

	**SGA, n (%)**	**OR**	**CI**	***P***
Low WC/low VF/low SCF (ref.)	63 (9.6)	1.00		
Low WC/high VF/low SCF	9 (7.5)	0.72	0.34 to 1.53	0.398
Low WC/low VF/high SCF	9 (9.1)	0.97	0.45 to 2.10	0.946
Low WC/high VF/high SCF	3 (10.0)	1.01	0.28 to 3.59	0.991
High WC/low VF/low SCF	2 (2.6)	0.43	0.10 to 1.87	0.259
High WC/high VF/low SCF	4 (7.4)	1.24	0.39 to 4.00	0.714
High WC/low VF/high SCF	12 (12.5)	1.92	0.75 to 4.93	0.176
High WC/high VF/high SCF	6 (5.6)	0.82	0.26 to 2.56	0.735

SGA, small-for-gestational-age; low WC <88 cm; high WC ≥88 cm; low VF <54 mm; high VF ≥54 mm; low SCF <21 mm; high SCF ≥21 mm.
WC, waist circumference; VF, visceral fat depth; SCF, subcutaneous fat depth; OR, odds ratio; CI 95% confidence interval
Data were analyzed using logistic regression.

The analysis was adjusted for maternal weight at the first antenatal visit (kg), maternal height (cm), parity (nulliparous or parous), smoking at the first antenatal visit (yes or no), maternal country of birth (EU or outside EU), pregestational diabetes (yes or no), and chronic hypertension (yes or no).

Supplementary Table S4. Association of the combination of WC and fat depth measures with SGA defined as birth weight standard deviation score <3rd percentile.

	**SGA, n (%)**	**OR**	**CI**	***P***
Low WC/low VF/low SCF (ref.)	19 (2.9)	1.00		
Low WC/high VF/low SCF	2 (1.7)	0.53	0.12 to 2.34	0.400
Low WC/low VF/high SCF	2 (2.0)	0.68	0.15 to 3.08	0.616
Low WC/high VF/high SCF	2 (6.7)	2.26	0.47 to 11.04	0.312
High WC/low VF/low SCF	1 (1.3)	0.60	0.07 to 4.95	0.632
High WC/high VF/low SCF	1 (1.9)	0.75	0.08 to 6.84	0.801
High WC/low VF/high SCF	2 (2.1)	0.82	0.13 to 5.19	0.835
High WC/high VF/high SCF	3 (2.8)	0.99	0.17 to 5.87	0.992

SGA, small-for-gestational-age; low WC <88 cm; high WC ≥88 cm; low VF <54 mm; high VF ≥54 mm; low SCF <21 mm; high SCF ≥21 mm.
WC, waist circumference; VF, visceral fat depth; SCF, subcutaneous fat depth; OR, odds ratio; CI 95% confidence interval
Data were analyzed using logistic regression.

The analysis was adjusted for maternal weight at the first antenatal visit (kg), maternal height (cm), parity (nulliparous or parous), smoking at the first antenatal visit (yes or no), maternal country of birth (EU or outside EU), pregestational diabetes (yes or no), and chronic hypertension (yes or no).
